# Effect of Preparation Methods on the Interface of LiBH_4_/SiO_2_ Nanocomposite Solid Electrolytes

**DOI:** 10.1021/acs.jpcc.4c02667

**Published:** 2024-07-17

**Authors:** Sander F. H. Lambregts, Laura M. de Kort, Frederik Winkelmann, Michael Felderhoff, Peter Ngene, Ernst R. H. van Eck, Arno P. M. Kentgens

**Affiliations:** † Magnetic Resonance Research Center, Institute for Molecules and Materials, Radboud University, 6525AJ, Nijmegen, The Netherlands; ‡ Materials Chemistry and Catalysis, Debye Institute for Nanomaterials Science, Utrecht University, 3584CG, Utrecht, The Netherlands; § Department of Heterogeneous Catalysis, Max-Planck-Institut für Kohlenforschung, 45470, Mülheim an der Ruhr, Germany

## Abstract

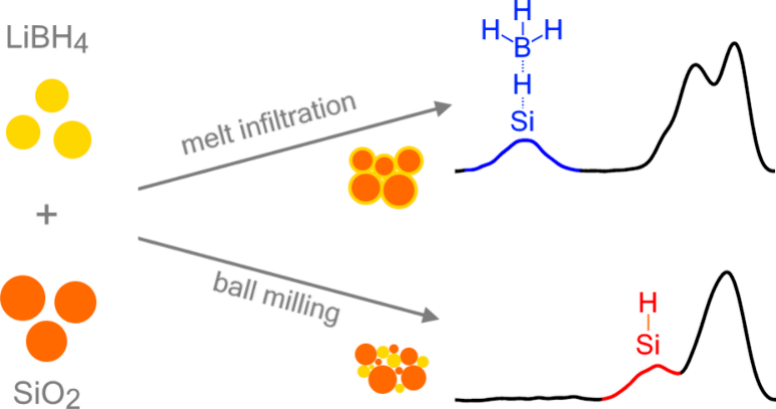

Nanocomposites of complex metal hydrides and oxides are promising solid state electrolytes. The interaction of the metal hydride with the oxide results in a highly conducting interface layer. Up until now it has been assumed that the interface chemistry is independent of the nanoconfinement method. Using ^29^Si solid state NMR and LiBH_4_/SiO_2_ as a model system, we show that the silica surface chemistry differs for nanocomposites prepared via melt infiltration or ball milling. After melt infiltration, a Si···H···BH_3_ complex is present on the interface, together with silanol and siloxane groups. However, after ball milling, the silica surface consists of Si– H sites, and silanol and siloxane groups. We propose that this change is related to a redistribution of silanol groups on the silica surface during ball milling, where free silanol groups are converted to mutually hydrogen-bonded silanol groups. The results presented here help to explain the difference in ionic conductivity between nanocomposites prepared via ball milling and melt infiltration.

## Introduction

The transition from energy sources based on fossil fuels to renewables has boosted research into energy storage technologies, including all-solid-state batteries. Compared to traditional Li-ion batteries that contain a liquid electrolyte, all-solid-state Li-ion batteries have demonstrated significant improvements in terms of safety, stability, and energy density.^1,2^

One class of electrolytes under investigation for use in all-solid-state batteries comprises the complex metal hydrides.^3−5^ Complex metal hydrides show good compatibility with high energy density anodes, including metallic lithium.^3,5^ In addition, they display good electrochemical stability, have a negligible electronic conduction, and are lightweight.^3,5^ One complex metal hydride that has received considerable interest is lithium borohydride. At elevated temperatures, the lithium ion of LiBH_4_ is highly mobile, resulting in a high ionic conductivity.^6^ Unfortunately, the ionic conductivity of bulk LiBH_4_ is poor below the structural phase transition from the orthorhombic to hexagonal phase (around 110 °C). Various strategies have been proposed to improve the ionic conduction at room temperature, for instance, by partial ionic substitution or by the use of other borohydride-like anions.^4,5,7,8^

A different strategy to increase the room temperature ion conductivity of complex metal hydrides involves the formation of nanocomposites, specifically bringing the hydride in contact with an oxide, such as silica.^9−12^ This approach has been shown to increase the conductivity of LiBH_4_/SiO_2_ by at least 3 orders of magnitude at ambient temperatures.^9,13^ It was revealed that the ion mobility at the interface between the oxide and the metal hydride was greatly enhanced.^12,14−17^ In melt-infiltrated LiBH_4_/(porous) SiO_2_ nanocomposites, the formation of Si···H···BH_3_ complexes and Li^+^ exchange with protons of silanol (SiOH) sites plays a pivotal role in the ionic conductivity increase.^18,19^

Nanocomposites of complex metal hydrides and oxides are most commonly prepared via two strategies: ball milling or melt infiltration. Ball milling, or mechanosynthesis, is based on high-energy impacts on the mixture to induce interface contacts. Melt infiltration on the other hand relies on wetting of the molten metal hydride on the surface of an oxide to form this interface. Previous comparative studies have revealed that both methods induce a significant improvement of the ionic conductivity, for example, Choi et al. reported an increase of 4 orders of magnitude for melt-infiltration and 3 orders of magnitude for ball milling.^13^

Up to this date, it has been assumed that the mechanism underlying the high ionic conductivity at the oxide/complex metal hydride interface is independent of the synthesis method. However, systematic studies have not yet been undertaken. In the present study we compare the interface interactions of nanocomposites prepared via ball milling or melt infiltration, making use of LiBH_4_/SiO_2_ nanocomposites as a model system. Solid-state NMR is used as the primary tool to probe the local environments of the isotopes at the interface between LiBH_4_ and the silica. We expect that the results of this study, which reveal a distinct difference between melt-infiltration and ball milling, can be generalized to other nanocomposites of oxides and complex metal hydrides, and their derivatives.

## Methods

### Sample Preparation

Fumed silica Aerosil 380 (AS) was obtained from Evonik. The synthesis and physical characterization of calcined mesoporous silica SBA-15, using a hydrothermal treatment temperature of 120 °C, is described by de Kort et al.^20^ Both silicas were dried at 300 °C for 6 h in a flow of 30 mL/min N_2_. This drying pretreatment resulted in the highest ionic conductivity in melt infiltrated LiBH_4_/SiO_2_ nanocomposites.^21^ All subsequent handling occurred in inert atmosphere in gloveboxes; tools and equipment were dried prior to sample contact.

Nanocomposites by melt infiltration (MI) were prepared according to the procedure by Ngene et al.^22^ Dried silica and LiBH_4_ were ground manually in a 1:1 weight ratio. The mixtures were transferred to glass vials which were placed in a stainless steel autoclave (Parr 4790, 50 mL) using PTFE gaskets. Approximately 50 bar H_2_ was added, and the autoclave was heated to 300 °C for 30 min using a ramp of 3 °C/min.

Ball milled (BM) nanocomposites were prepared by mixing dried silica and LiBH_4_ in a 1:1 weight ratio. The sample and 16 10 mm stainless steel balls (ball/sample weight ratio of about 220:1) were added to a stainless steel (1.4571, 50 mL) autoclave. Either 50 bars H_2_ or 1 bar Ar was added to the autoclaves. The ball mill (Fritsch Pulverisette 6) was operated at 500 rpm for 1 h, reversing the direction every 10 min.

The nanocomposites are referred to by their preparation method (MI, BM(Ar) or BM(H_2_)) and the used silica (AS or SBA), e.g., BM(Ar)-SBA is a LiBH_4_/SBA-15 nanocomposite prepared by ballmilling in argon.

The dried silica scaffold used to study the effect of ball milling without LiBH_4_ was ball milled in a dry N_2_ atmosphere using 7 15 mm ZrO_2_ balls (ball/sample weight ratio about 380:1) in a ZrO_2_ cup on a Retsch PM100 ball mill operating at 400 rpm for 1 h, reversing the direction every 10 min. Based on introductionary experiments preparing LiBH_4_/SiO_2_ nanocomposites in the ZrO_2_-based milling equipment compared to samples prepared using stainless steel balls shown here, different grinding media composition are not expected to influence the results.

### Solid-State NMR Measurements

Solid-state NMR experiments were performed on 7.05 and 9.39 T Varian VNMRS, and 22.3 T Bruker AVANCE III HD spectrometers using Bruker 4.0 mm MAS (7.05 T), Varian 3.2 mm T3 and RevolutionNMR 6.0 mm MAS (9.39 T), and Bruker 3.2 mm (22.3 T) probes, respectively. All experiments were performed in a flow of N_2_, and sample preparation was done in a N_2_-filled glovebox.

^29^Si spectra were all measured using cross-polarization (CP)^23^ and Carr–Purcell–Meiboom–Gill (CPMG)^24,25^ detection consisting of a train of 180° pulses, except for the ^29^Si{^11^B} REDOR (no CPMG). Six to 10 echoes were used for CPMG. The rotational-echo, double-resonance (REDOR)^26^ experiments were recorded using a single 180° pulse on ^29^Si, and a train of two 180° pulses per rotor period on ^7^Li or ^11^B. Experiments utilizing CP under Lee–Goldburg conditions (LGCP)^27,28^ used the ±2 Hartmann–Hahn matching condition. For the ^1^H spectrum of BM(Ar)-AS, the DEPTH background suppressing sequence was used.^29^ The two-dimensional heteronuclear correlation spectrum utilized the wide-line separation sequence^30^ with LGCP and CPMG. ^11^B single pulse excitation (SPE) experiments employed 25–30° excitation pulses, all other direct excitation experiments used 90° pulses. All experiments utilized radiofrequency (RF) field strengths between 40 and 60 kHz, with the exception of ^1^H SPE and DEPTH experiments (60–75 kHz), ^6^Li (33 kHz) and ^11^B SPE experiments (65–110 kHz). SPINAL^31^^1^H decoupling with RF field strengths of 30–100 kHz was used in all except ^1^H-detected experiments. Decoupling was also used during CPMG detection and REDOR evolution.

Spectra were processed with ssNake^32^ and referenced to adamantane (δ(^13^C) = 38.5 ppm), zeolite A (δ(^29^Si) = −89.7 ppm), methanol (δ(^1^H) = 3.3 ppm), or aqueous LiCl (δ(^6,7^Li) = 0.0 ppm), using the frequency ratios if needed.^33−35^ REDOR curves were fitted to the formula of Hirschinger.^36^ LGCP build-up curves were fitted to the formula of Hediger;^37^ the dipolar interaction herein was scaled by a factor  to correct for the ±2 CP matching condition and the Lee–Goldburg scaling.

## Results

### Effect of Preparation Method on Silica

**Figure 1:**
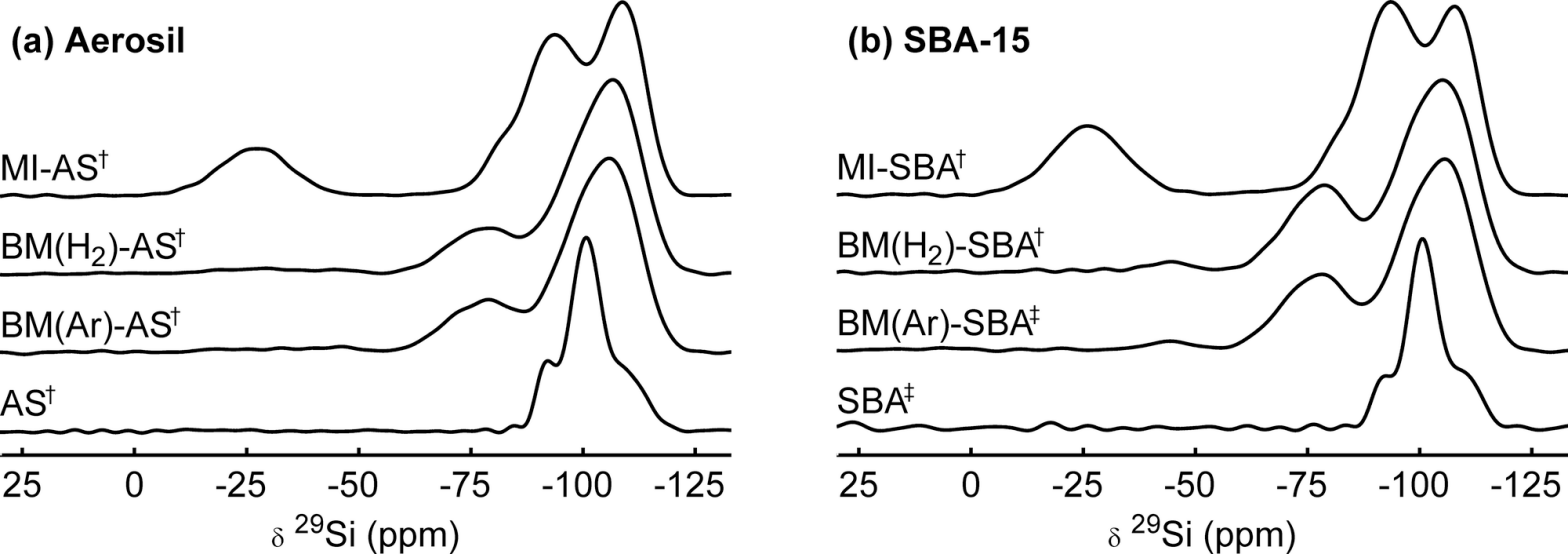
^29^Si CP spectra of LiBH_4_/SiO_2_ nanocomposites prepared via melt infiltration and ball milling using (a) Aerosil and (b) SBA-15 as silica scaffold, and the corresponding silica scaffolds without LiBH_4_ (lower trace). A contact time of 2 ms was used, at a spinning speed of 6.25 kHz in a field of 7.05 (‡) or 9.4 T (†), with CPMG acquisition. Deconvolution of the peaks in nanocomposites prepared via ball milling can be found in Figure S1.

Figure 1 shows the ^29^Si NMR spectra of LiBH_4_/SiO_2_ nanocomposites prepared via melt infiltration or ball milling, and their silica scaffold. Surprisingly, there are significant differences between the spectra of the nanocomposites. The spectra of the silica scaffolds (without LiBH_4_, dried at 300 °C) each show, in accordance with literature,^38,39^ three partially overlapping peaks at −110, −101, and −91 ppm. These correspond to Q_*n*_ sites with *n* = 4, 3 or 2, respectively, where Q_*n*_ refers to a silicon site with *n* bridging oxygen atoms and 4 – *n* hydroxyl groups, e.g., Si (O–Si−)_*n*_(OH)_4–*n*_. Due to the relatively mild drying conditions of the silica, silanol sites (Q_2,3_) are still present.^40^ These drying pretreatment conditions result in the optimal ionic conductivity for melt-infiltrated LiBH_4_/SiO_2_ nanocomposites.^21^

The spectra of melt-infiltrated LiBH_4_/SiO_2_ show good resemblance to the spectra shown in our recent work.^18^ Compared to the silica scaffold, an extra peak is observed around −25 ppm. We attributed this peak to a Si···H···BH_3_ complex that is formed during melt infiltration, which we refer to as the melt-infiltration-induced (mii) site. Additionally, the Q_3_ peak shifts to −94 ppm upon melt infiltration, , which we attributed to exchange of lithium ions with protons of the silanol groups.

Interestingly, the ^29^Si NMR spectra of LiBH_4_/SiO_2_ nanocomposites prepared via ball milling show distinct differences compared to the aforedescribed ^29^Si spectra of the silicas and the nanocomposites prepared via melt infiltration. They consist of two overlapping peaks at −109 and −101 ppm, and overlapping peaks at −81 and −73 ppm. The chemical shifts of the peaks at −109 and −101 ppm match very well with the shifts of Q_4_ (siloxane) and Q_3_ (silanol) sites, respectively, and are therefore assigned as such. In contrast, overlapping resonances at −73 and −81 ppm have not been described in literature for a system similar to ball-milled LiBH_4_/SiO_2_ before.

The ^29^Si NMR spectra of the nanocomposites prepared via ball milling under Ar (atmospheric pressure) or H_2_ (50 bar) are nearly identical, indicating that the ball milling atmosphere does not have a significant influence on the interface interactions between LiBH_4_ and silica. This is in stark contrast to melt infiltration, where the use of H_2_ is crucial to avoid decomposition of the nanocomposite.^22^

**Figure 2:**
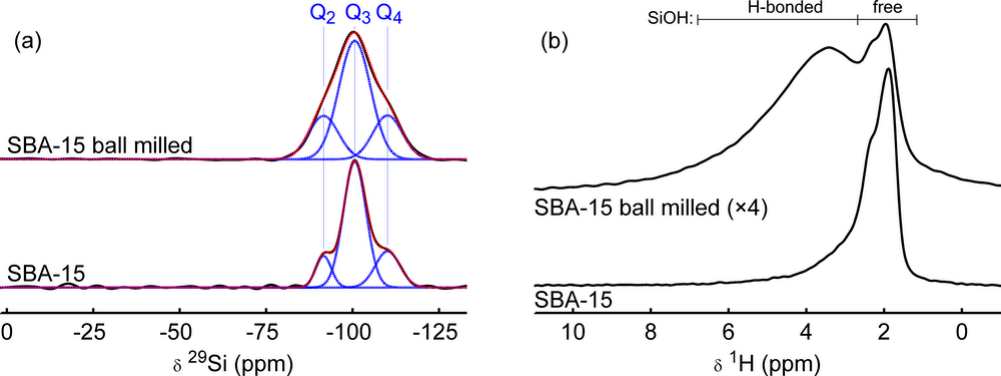
(a) ^29^Si CP and (b) ^1^H SPE NMR spectra of the dried silica SBA-15, without LiBH_4_, before and after ball milling. All spectra were acquired on a 7.05 T magnet under 6.25 kHz MAS. The ^29^Si spectra were recorded using CPMG acquisition and a contact time of 2 ms. The ^29^Si spectrum consists of 3 peaks: Q_2_, Q_3_, and Q_4_ (Q_*n*_ = Si (O–Si−)_*n*_(OH)_4–*n*_). Free silanol sites refer to silanol sites that have no (or only weak) hydrogen bonds to neighboring silanol sites, and includes isolated silanol sites. The intensity of the ^1^H spectrum of the silica after ball milling is multiplied by 4 compared to the ^1^H spectrum of the silica before ball milling to aid visual comparison.

To determine whether the resonances at −73 and −81 ppm originate from an interaction between LiBH_4_ and the silica scaffold, rather than being a consequence of ball milling of silica, a dried silica scaffold (SBA-15) was ball milled without LiBH_4_. Figure 2 shows the ^29^Si and ^1^H NMR spectra of this scaffold before and after ball milling. The ^29^Si spectra reveal that the Q_2_ and Q_3_ resonances have broadened after ball milling, and the relative intensity of the Q_2_ resonance has increased at the expense of the intensity of the Q_3_ resonance. The ^1^H spectrum (Figure 2b) before ball milling only shows resonances around 2 ppm, whereas after ball milling the intensities of these peaks have decreased, and a large broad peak centered around 4 ppm is observed instead. The resonances around 2 ppm are usually associated with free (non-hydrogen bonded) and weakly hydrogen-bonded silanol sites, whereas the broad resonance corresponds to silanol sites in various hydrogen-bonding environments.^41−43^ Both the ^29^Si and ^1^H spectra thus indicate that silanol sites are redistributed during ball milling of the dried silica scaffold, favoring a hydrogen-bonded silanol network over free silanol sites. However, as the ^29^Si spectra do not reveal new sites after ball milling, the sites at −73 and −81 ppm in nanocomposites prepared via ball milling cannot originate from ball milling of the silica and must originate from an interaction between LiBH_4_ and the silica scaffold.

Unlike the spectra of melt-infiltrated LiBH_4_/SiO_2_, the spectra of the ball-milled nanocomposites shows no peak around −25 ppm. In addition, the Q_3_ resonance has not shifted compared to the Q_3_-site of the parent silica. Concurrently, the spectra of the melt-infiltrated nanocomposites show no resonance around −73 ppm. These differences between the ^29^Si spectra of nanocomposites prepared via melt infiltration or ball milling reveal that the surface interactions between LiBH_4_ and silica differ depending on the synthesis method used.

### Interaction between LiBH_4_ and Silica after Ball Milling

**Figure 3:**
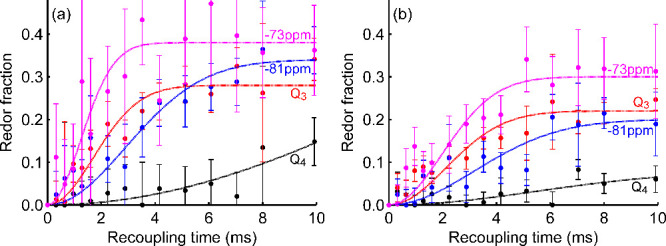
^29^Si{^7^Li} REDOR difference curves of the nanocomposites (a) BM(Ar)-AS and (b) BM(H_2_)-SBA. The data were acquired at 9.4 T under 6.25 kHz MAS. The experiment utilized polarization transfer from ^1^H to ^29^Si. The solid lines are the least-squares fits to the formula of Hirschinger;^36^ the fit results can be found in Table S1. The error bars indicate the ±1 standard deviation. Slices of the REDOR experiment can be found in Figure S2.

Figure 3 shows the REDOR difference curves of the resonances in LiBH_4_/SiO_2_ nanocomposites prepared via ball milling. A REDOR experiment probes the dipolar couplings between isotopes (^7^Li and ^29^Si in this experiment), which is proportional to the *r*^–3^-weighted average through-space distance between these isotopes. A fast build-up is indicative of a strong dipolar coupling and a close proximity of the isotopes. The REDOR curves corresponding to the sites at −73 and −81 ppm and the Q_3_-site have comparable build-up rates, with the exception of the curve for the site at −73 ppm in BM(Ar)-AS. The latter exception may however be the result of the large error margins, which would also explain the difference between the two nanocomposites. Fitting of the REDOR curves of the Q_3_ site and the sites at −73 and −81 ppm yields *r*^–3^-weighted average Li–Si distances between 3.3 and 4.6 Å (Table S1).

None of the REDOR curves approaches a REDOR fraction of 0.92, the natural abundance of ^7^Li. Reaching 0.92 or higher would imply that all ^29^Si atoms of a certain species participate in an interaction with lithium. Instead, the curves of the sites at −73 and −81 ppm and Q_3_ flatten around 0.3. This suggests that only roughly a third of these sites are in the proximity of lithium; the remainder of the silicon sites has no lithium in its proximity. The Q_4_ site could not be fitted well, but either has a very slow build-up, or levels off close to 0, both suggesting that it does not have significant interaction with lithium.

**Figure 4:**
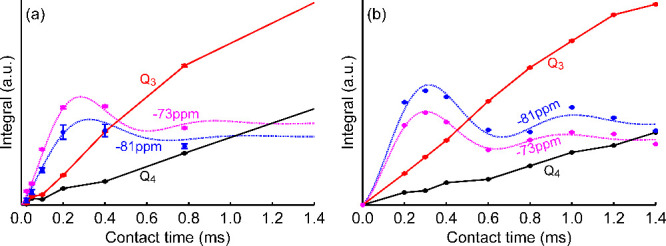
{^1^H}^29^Si LGCP build-up curve of the nanocomposites (a) BM(Ar)-AS and (b) BM(H_2_)-SBA. The data for both curves was measured at 9.4 T under 6.25 kHz MAS. Both experiments were acquired using CPMG. The dashed lines are least-squares fits of the dipolar oscillations of the sites resonating at −73 and −81 ppm, which are fitted to the formula of Hediger.^37^ The solid lines connect the data points for the Q_3_ and Q_4_ resonances to guide the eye. LGCP build-up curves with longer contact time can be found in Figure S3.

We also probed the dipolar coupling strength between ^1^H and ^29^Si using Lee–Goldburg cross-polarization (LGCP) experiments.^27^Figure 4 shows the buildup of magnetization on ^29^Si from ^1^H as a function of the cross-polarization time. This build-up is proportional to the average *r*^–3^-weighted distance between ^1^H and ^29^Si.

The LGCP build-up curves of the sites resonating at −73 and −81 ppm both display oscillating behavior at short cross-polarization times. This is characteristic for ^29^Si sites interacting with an isolated proton, located at a constant distance from each other. The oscillation frequency is directly proportional to the dipolar interaction between ^1^H and ^29^Si.^37,44^ The strength of the dipolar interaction could be determined via fitting as 5.3 ± 0.2 and 4.9 ± 0.3 kHz for the sites at −73 and −81 ppm, respectively, after correcting for the LG and CP-MAS conditions. For an isolated silicon-proton pair, this corresponds to a proton-silicon distance of 1.7 ± 0.1 Å. A distance of 1.7 Å is significantly shorter than the through-space distance between silicon and proton in a silanol group (about 2.3 Å) but only slightly longer than the expected bond length in a SiH group (about 1.5 Å), which might be due to the uncertainty in the scaling factor of the LG irradiation.

In contrast to the sites at −73 and −81 ppm, the polarization build-up for the Q_3_ and Q_4_ sites is more gradual and devoid of oscillations, which is usually indicative of a pool of spins coupling to the same nucleus.

**Figure 5:**
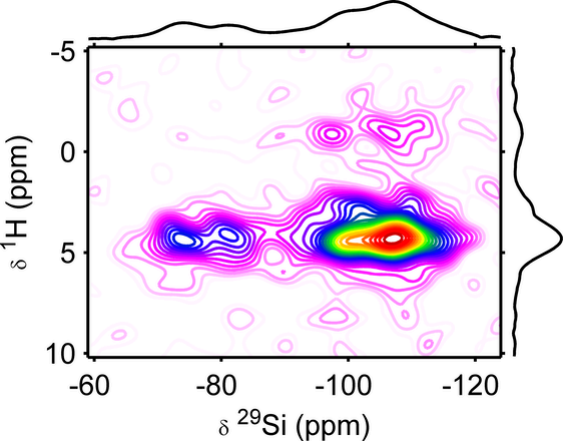
{^1^H}^29^Si heteronuclear correlation spectrum of BM(H_2_)-AS, measured at 9.4 T under 6.25 kHz MAS with LGCP polarization transfer (contact time: 2 ms) and CPMG acquisition. Slices can be found in Figure S4.

LGCP build-up curves do not reveal which proton (e.g., , SiOH, ...) is coupled with each silicon site. This information can however be derived from a 2-dimensional heteronuclear correlation spectrum as shown in Figure 5, which reveals which ^1^H site couples to which ^29^Si site. The Q_4_ and Q_3_ sites of the silica (at δ(^29^Si) = −109 and −101 ppm, respectively) correlate with protons resonating at 4 and −1 ppm. The resonance at 4 ppm corresponds to the ^1^H resonance of silanol sites (Figure 2) and is thus expected to couple with Q_3_ sites and sites in close proximity to Q_3_ sites. The ^1^H chemical shift at −1 ppm matches the ^1^H chemical shift of LiBH_4_. This correlation between LiBH_4_ and the Q_3_ and Q_4_ sites reveals that there are  ions in the proximity of the silica. This is in line with our previous findings on melt infiltrated LiBH_4_/SiO_2_. The intensity of the LiBH_4_ correlation peak is much smaller than in a direct-excitation ^1^H NMR experiment (Figure S5), which is expected in case the LiBH_4_ is mobile, at a large average distance, or only a small fraction of the LiBH_4_ interacts with the silica. This is also reflected in the ^29^Si{^11^B} REDOR experiment (Figure S6), which only reveals weak interactions between boron and silicon.

The ^29^Si resonances at −73 and −81 ppm also couple with a ^1^H peak at 4 ppm. However, unlike the Q_3_ and Q_4_ resonances, no correlation of these sites with the proton peak of LiBH_4_ is observed, suggesting that there are no  in the vicinity of the sites resonating at −73 and −81 ppm. This is also corroborated by the ^29^Si{^11^B} REDOR (Figure S6), which does not show strong interactions between boron and silicon and by the LGCP build-up (Figure 4) that reveals that the proton is isolated from other protons.

### Majority of LiBH_4_ Remains Intact

The differences in the ^29^Si NMR spectra of melt-infiltrated and ball-milled nanocomposites reveal a difference in the interaction between lithium borohydride and silica. In order to verify whether LiBH_4_ is still intact, ^11^B NMR spectra were recorded.

**Figure 6:**
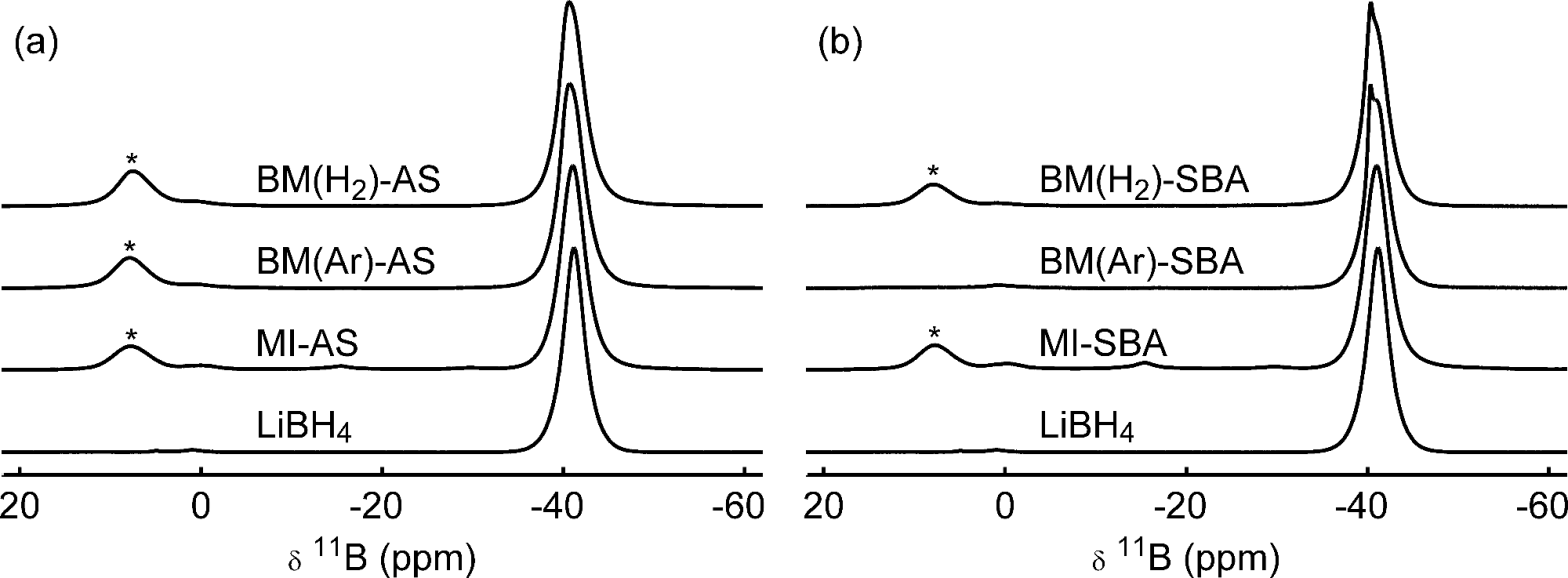
Normalized ^11^B MAS NMR spectra of bulk LiBH_4_ and nanocomposites using Aerosil (a) or SBA-15 (b), prepared using ball milling in Ar or H_2_ or via melt infiltration. The spectra were measured at 9.4 T under 6.25 or 10.0 kHz MAS. Spinning sidebands for the spectra measured at 6.25 kHz MAS are indicated by asterisks.

The largest peak in the ^11^B spectra of the nanocomposites, shown in Figure 6, consists of two overlapping resonances around −40 and −41 ppm. These resonances are typically assigned to highly dynamic and less dynamic (bulk-like) LiBH_4_, respectively.^14^ Aside from the differences in the relative intensities of these two peaks, which may reflect the effect of the milling efficiency with the different silica scaffolds (out of the scope of this manuscript), the spectra do not reveal significant differences in this spectral region. The similarity of the ^11^B spectra of the nanocomposites, together with the ^1^H chemical shift of −1 ppm (Figures 5 and S5), and a ^6^Li spectrum of a ball-milled nanocomposite that highly resembles a melt-infiltrated nanocomposite (Figure S7), show that the LiBH_4_ remains mostly intact.

A commonly proposed model is that Si–O–B species are formed on the interface between LiBH_4_ and silica.^16,45^ If such species are abundantly present, their resonances should be observable in the ^11^B NMR spectra. However, the ^11^B NMR spectra of nanocomposites prepared via ball milling do not reveal any resonances other than those corresponding to  near −41 ppm, and some (very low intensity; see Figure S8 for a magnified view) peaks between 10 and 0 ppm, which are already present in bulk LiBH_4_. This is unlike the spectra of nanocomposites prepared via melt infiltration, where also small peaks are observed at approximately −2, −15, and −30 ppm (see also Figure S8), which we ascribed to partial decomposition of the LiBH_4_ in our earlier study.^17^ Although trace amounts cannot be excluded (vide infra), the near-absence of boron resonances in spectra of nanocomposites prepared via ball milling, other than LiBH_4_ and the impurity in bulk LiBH_4_, contrasts the model that Si–O–B species are abundantly formed.

## Discussion

The ^29^Si NMR spectra in this paper demonstrate that the interaction between lithium borohydride and silica highly depends on whether the nanocomposite was prepared via ball milling or melt infiltration. Consequently, the existing interface model for nanocomposites prepared via melt infiltration^18^ cannot be applied to nanocomposites prepared via ball milling.

### Chemical Nature of the New Sites

The resonances at δ(^29^Si) = −73 and −81 ppm in nanocomposites prepared via ball milling have a strong interaction with proton and only a negligible interaction with boron. This further rules out that they correspond to sites similar to the Si···H···BH_3_ (“*mii*”) complex observed in nanocomposites prepared via melt infiltration, as this *mii* complex has a much stronger silicon–boron interaction. Three alternative scenarios for the origin of the resonances at δ(^29^Si) = −73 and −81 ppm are explored: whether these resonances correspond to silanol groups, lithium silicates, or silicon hydrides. Silanol groups and silicon hydride bridges are crucial in nanocomposites prepared via melt infiltration; and lithium silicates are a possible decomposition product of LiBH_4_ and silica.^18,22^

It is however unlikely that the resonances at δ(^29^Si) = −73 and −81 ppm correspond to silanol sites. The presence of oscillations in the LGCP build-up curve excludes hydrogen bonded silanol sites, whereas free Q_2_ or Q_3_ silanol sites are expected to resonate between −90 to −102 ppm in the ^29^Si spectrum and around 2 ppm in the ^1^H spectrum.^43^ In addition, the through-space Si-to-H distance of 1.7 Å (for a single proton interacting with the silicon) excludes Q_3_ sites, as this distance would only be achieved if the Si–O–H bond angle is distorted to about 75°.

Also unlikely is the scenario that these peaks correspond to pure lithium silicates. Although Q_2_ groups of lithium silicates can resonate around −76 ± 2 ppm,^46,47^ close to the observed chemical shifts, those Q_2_ sites of lithium silicates are in close proximity to lithium.^48^ Our REDOR experiment (Figure 3), however, shows that less than half of the resonances at δ(^29^Si) = −73 and −81 ppm is in the proximity of lithium. In addition, the presence of just lithium silicates would not explain the strong silicon–proton interaction.

More probable is that the resonances at δ(^29^Si) = −73 and −81 ppm correspond to silicon hydride (SiH) sites. Both silicon resonances have a distinct dipolar interaction with ^1^H, which matches the interaction expected for a single proton at a distance of 1.7 Å from silicon. This proton–silicon distance is only slightly longer than a typical Si–H bond length (about 1.5 Å). In addition, the observed ^29^Si and ^1^H chemical shifts are very close to the expected chemical shifts of silicon hydrides, e.g., δ(^29^Si) = −72 and −82 for H–Si(OH)O_2_= and H–SiO_3_≡, respectively, and a ^1^H resonance around 4 ppm.^49−51^ Consequently, we assign the resonances at δ(^29^Si) = −73 and −81 ppm to silicon hydrides.

The observation of oscillations in the LGCP build-up curve indicates that the Si–H distance is very constant on the millisecond time scale. Consequently, it is unlikely that these sites are directly involved in chemical exchange processes. This is in stark contrast to the *mii* site in melt infiltrated nanocomposites, where the Si···H···BH_3_ complex constantly exchanges with .

### Origin of the Difference between Ball Milling and Melt Infiltration

Of particular interest is what causes the difference in the interface interaction for nanocomposites prepared via ball milling or melt infiltration. While ball milling is commonly performed in inert atmospheres, melt infiltration of LiBH_4_ is performed in a pressurized H_2_ atmosphere. The use of H_2_ during melt infiltration is crucial to avoid decomposition of the nanocomposite at elevated temperatures.^22^ Therefore, we studied the effect of the atmosphere during ball milling. Our results demonstrate that the use of an Ar or pressurized H_2_ atmosphere during ball milling at ambient temperature does not result in notable differences in the ^29^Si spectra. Consequently, we cannot ascribe the difference on the LiBH_4_–silica interface between nanocomposites prepared via melt infiltration or ball milling to the atmosphere used during their synthesis.

Another hypothesis is that a heating cycle is necessary to induce the conversion of SiH to *mii* sites. Electrochemical impedance spectroscopy studies found that the conductivity of nanocomposites prepared via ball milling increases after a heating cycle to above the structural phase transition of LiBH_4_.^11^ However, exposing a nanocomposite prepared via ball milling to temperatures above this phase transition did not result in changes in the NMR spectra (Figure S9). More likely, the aforementioned effects of a heating cycle are related to the annealing of defects in LiBH_4_.^11,52^ Hence, we exclude that a heating cycle (below the melting or decomposition temperature, but above the structural phase transition) induces the differences on the LiBH_4_–silica interface between melt-infiltrated and ball-milled nanocomposites.

A possible explanation for the differences between nanocomposites prepared via ball milling and melt infiltration can be found in the silanol region. Free silanol groups, silanol groups that are not hydrogen-bonded to other groups, are crucial for the high Li^+^ conductivity in nanocomposites prepared via melt infiltration.^18,21^ However, during ball milling, the vast majority of the free silanol groups of the silica in the nanocomposite is converted to silanol groups that are hydrogen bonded to neighboring silanol groups, as reflected by the ^1^H resonance around 4 ppm in the 2-dimensional spectrum (Figure 5), rather than around 2 ppm, as expected for free silanol groups.^41−43^ This redistribution of silanol groups due to ball milling can also be observed in a ball milled silica scaffold without LiBH_4_ (Figure 2) and is likely related to breakage of Si–O bonds during ball milling,^53^ in combination with the predrying of the silica scaffolds at 300 °C.

As the redistribution from free to hydrogen bonded silanol groups occurs readily during the ball milling process, it is likely that this plays a major role in the interaction between LiBH_4_ and silica. Possibly the lower availability of free silanol sites acts as a barrier for the formation of a stable Si···H···BH_3_ complex and reduces the SiOH/SiOLi-exchange that is observed for nanocomposites prepared via melt infiltration. In that case, it may be necessary to further reduce the initial silanol concentration of the silica prior to ball milling, to make it less likely that silanol groups can form a hydrogen-bonded silanol network, thus, retaining a high amount of the active, free silanol groups.

The remaining question is what is the origin of the SiH sites. As additional H atoms are introduced in the silica material these most realistically originate from a reaction with . One possibility is that a small amount of the  anions reacts at the location of broken Si–O–Si bonds, similar to melt infiltration, where Si···H···BH_3_ complexes are formed after breakage of Si–O–Si bonds, but in this case dissociates into gaseous BH_3_, leaving behind the ≡SiH group. Another hypothesis explaining the formation of SiH sites is that LiBH_4_ or ···BH_3_, reacts with the hydrogen-bonded silanol groups and/or water from condensing silanol groups. Coordination of BH_3_ to silanol groups has been postulated for NH_3_BH_3_ by Lai et al.^54^ If such a reaction under ball milling conditions produces various compounds and these compounds are each formed in trace amounts, this could explain why they are not observed in ^11^B MAS NMR spectra (or, analogous to this, why no extra lithium resonances are observed in the ^6^Li NMR spectrum). These explanations remain speculative, however, as our NMR results study do not allow us to derive a definite mechanism for the formation of the SiH sites.

It is very likely that the difference in the interface interactions between nanocomposites prepared via ball milling and melt infiltration affects the ionic conductivity. Although comparison of the overall ionic conductivities is out of the scope of this manuscript, Choi et al. studied the difference in ionic conductivity of ball milled and melt infiltrated nanocomposites, and observed that the overall ionic conductivity of a nanocomposite prepared via ball milling was an order of magnitude lower compared to a nanocomposite prepared by melt infiltration.^13^ As the overall ionic conductivity also depends on other factors, such as defect formation in LiBH_4_^52,55^ and scaffold properties, it remains to be seen whether this difference in conductivity can be attributed to the differences in interface chemistry alone.

## Conclusions

We have studied the interface interactions between LiBH_4_ and silica for nanocomposites prepared via two common preparation methods: ball milling and melt infiltration. The results demonstrate that the interface chemistry is significantly different between the two preparation methods.

In nanocomposites prepared via melt infiltration, we have previously demonstrated and confirmed herein that siloxane bonds are broken to form an exchanging Si···H···BH_3_ complex (resonating at δ(^29^Si) ≈ −25 ppm) and a silanol site that exchanges with Li^+^ (δ(^29^Si) ≈ −94 ppm).

In nanocomposites prepared via ball milling, these effects are not observed. Instead, novel sites are observed, resonating around δ(^29^Si) ≈ −73 and −81 ppm, which are assigned to SiH sites. We propose that this interaction is the result of a rearrangement of the silanol sites during ball milling and a reaction with  anions. Apparently, the formation of a stable borohydride–hydride bridge complex, as observed for melt infiltrated samples, is prevented. We furthermore did not observe direct evidence for the formation of new B–O-bonded complexes, other than the impurities already present in bulk LiBH_4_.

The results in this manuscript reveal a previously unexpected difference between the synthesis methods of nanocomposites. These results improve the understanding of differences in the conductivity of nanocomposites prepared via different synthesis methods. Although the results were obtained using the model system LiBH_4_/SiO_2_, these may apply to all nanocomposites that are eligible for preparation using either technique.

## Supporting Information Available

Fit results of ^29^Si{^7^Li} REDOR curves; Deconvolution of ^29^Si NMR spectrum; Slices and extracts of REDOR curves and 2D correlation experiments; ^29^Si{^11^B} REDOR curves; LGCP build-up curves with longer contact times; Additional ^6^Li, ^1^H and ^29^Si MAS NMR experiments (PDF)
