# Supplementary material for: Effect of Preparation Methods on the Interface of LiBH4/SiO2 Nanocomposite Solid Electrolytes
Source: J Phys Chem C Nanomater Interfaces. 2024 Jul 17;128(29):12186–93. doi: 10.1021/acs.jpcc.4c02667 (PMC11284851; doi:10.1021/acs.jpcc.4c02667)
Supplement: Supplementary file 1 — jp4c02667_si_001.pdf [file jp4c02667_si_001.pdf]

Supporting Information: The Effect of Preparation Methods on the Interface of $LiBH_4/SiO_2$ Nanocomposite Solid Electrolytes

Sander F. H. Lambregts,[†] Laura M. de Kort,[‡] Frederik Winkelmann,[¶] Michael Felderhoff,[¶] Peter Ngene,[‡] Ernst R. H. van Eck,[†] and Arno P. M. Kentgens[*,†]

†*Magnetic Resonance Research Center, Institute for Molecules and Materials, Radboud University, 6525 AJ, Nijmegen, The Netherlands.*

‡*Materials Chemistry and Catalysis, Debye Institute for Nanomaterials Science, Utrecht University, 3584 CG, Utrecht, The Netherlands.*

¶*Department of Heterogeneous Catalysis, Max-Planck-Institut für Kohlenforschung, 45470, Mülheim an der Ruhr, Germany*

E-mail: a.kentgens@nmr.ru.nl

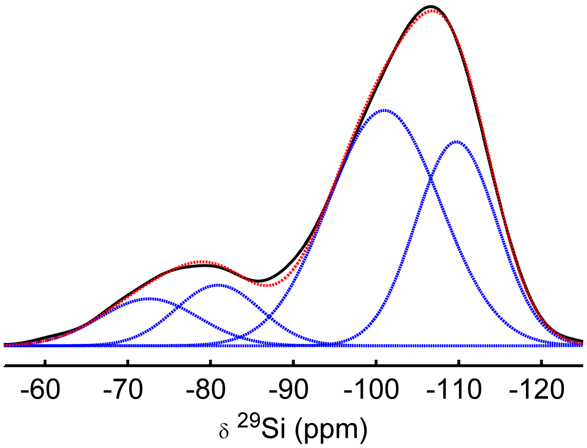

Figure S1: Peak deconvolution of the ^{29}Si spectrum of BM(H_2)-AS as shown in Figure 1a. Deconvolutions of melt-infiltrated nanocomposites can be found in our previous article.[S1]

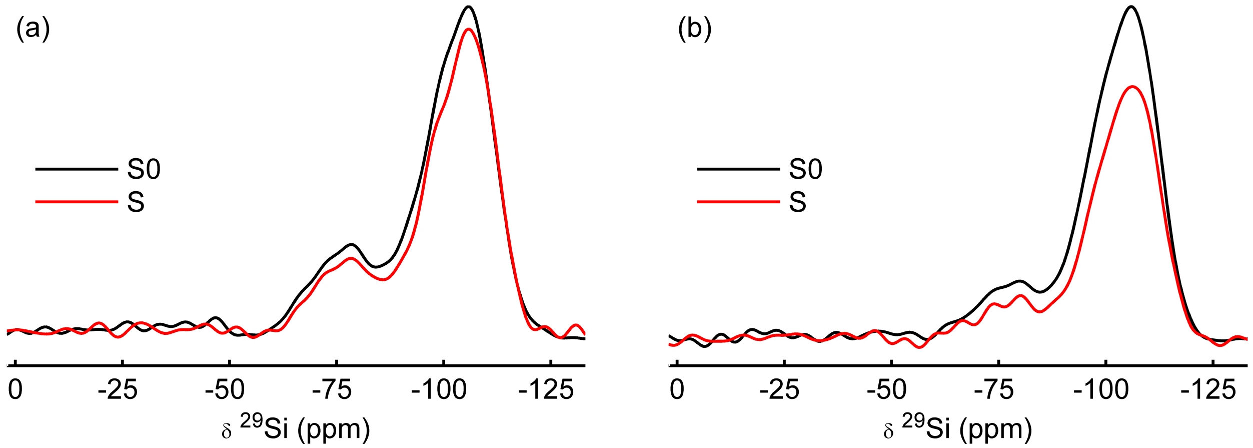

Figure S2: The spectra based on which REDOR fractions for BM(Ar)-AS at a recoupling time of (a) 2.88 and (b) 9.92 ms were calculated. The REDOR fraction is calculated as (S0 − S) ÷ S0. The full REDOR curve can be found in Figure 3a.

Table S1: Second moments and r^{-3}-weighted average through-space Li - Si distances obtained by fitting[S2] of the $^{29}Si\{^{7}Li\}$ REDOR curves shown in Figure 3. The fitting assumes that each ^{7}Li isotope is dipolar-coupled to one silicon atom of a certain species. The REDOR curve of the Q_4 site could not be fitted reliably in either nanocomposite and has therefore been excluded (ND) from the results. The fitting error in the weighted averaged distances is estimated as 0.5 Å.

Nanocomposite	Second moment (kHz^2)				Internuclear distance (Å)			
	−73 ppm	−81 ppm	Q_3	Q_4	−73 ppm	−81 ppm	Q_3	Q_4
BM(Ar)-AS	2.7	0.4	1.1	ND	3.3	4.4	3.8	ND
BM(H_2)-SBA	0.8	0.4	1.0	ND	4.0	4.6	3.9	ND

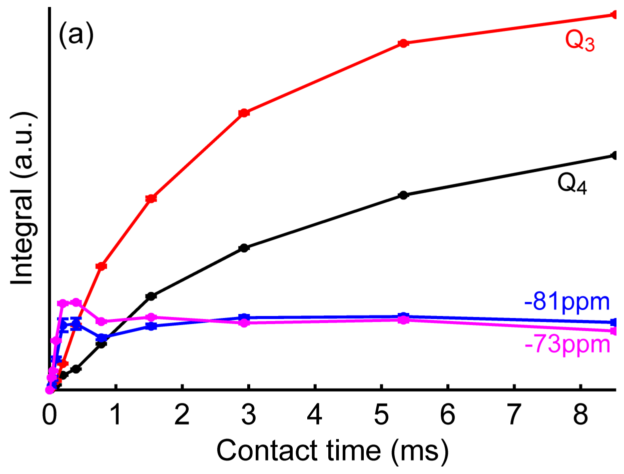

Figure S3: {^{1}H}^{29}Si LGCP build-up curve of the nanocomposites (a) BM(Ar)-AS and (b) BM(H_2)-SBA. The data for both curves was measured at 9.4 T under 6.25 kHz MAS. Both experiments were acquired using CPMG. The data in (b) was measured in a different experiment than Figure 4, resulting in small intensity differences due to different CP or LG conditions. Lines connecting the data points were added to guide the eye.

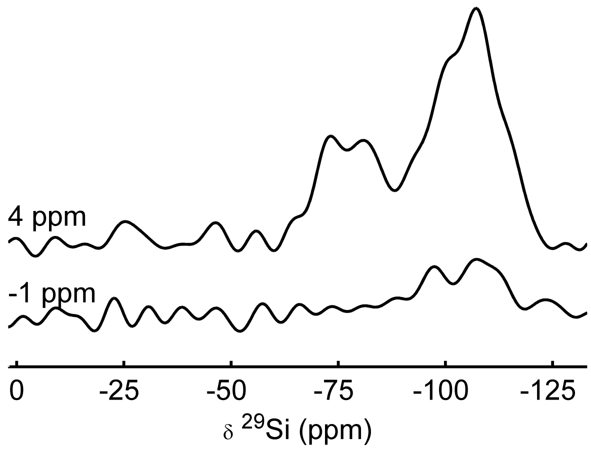

Figure S4: Horizontal slices of the 2-dimensional spectrum shown in Figure 5. The slices show the ^{29}Si dimension, while the spectrum label indicate the center position of the ^{1}H dimension where the slice was taken. Each slice is composed of the sum of all horizontal slices within ±1 ppm from the listed ^{1}H shift, i.e. the −1 ppm slice contains all intensity between $\delta(^1H) = -2$ to 0 ppm.

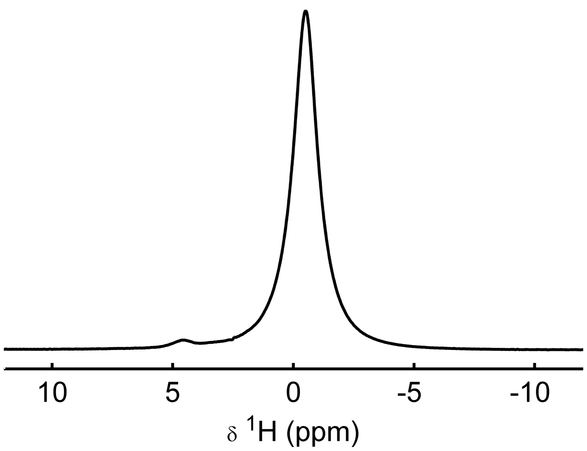

Figure S5: ^{1}H MAS NMR spectra of the nanocomposite BM(Ar)-AS, measured at 22.3 T under 18 kHz MAS using DEPTH.

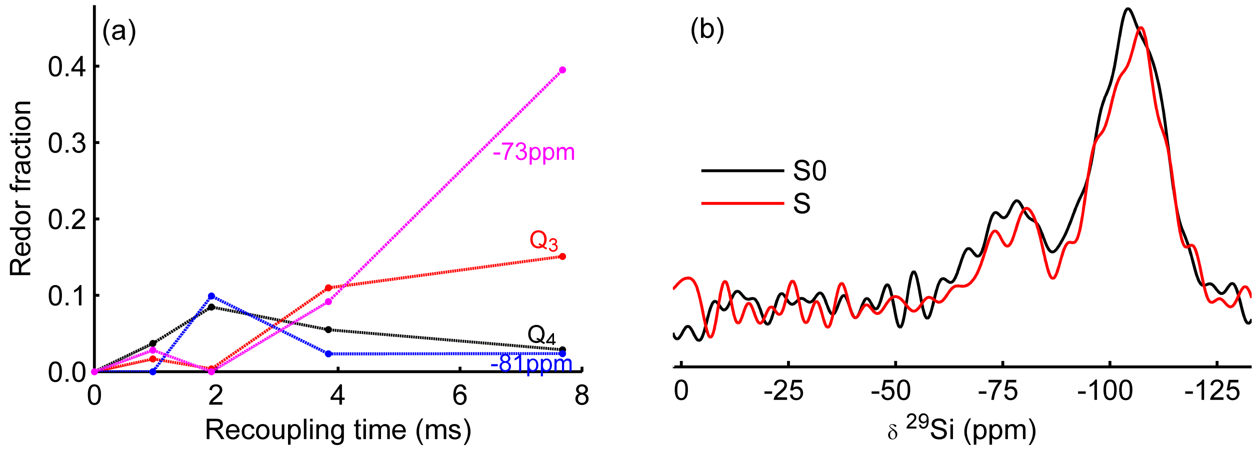

Figure S6: (a) ^{29}Si{^{11}B} REDOR difference curves of the nanocomposite BM(Ar)-AS. The data was measured at 9.4 T under 6.25 kHz MAS and utilized magnetization transfer from ^{1}H to ^{29}Si. The lines only connect the data points to guide the eye and have no physical meaning. The data could not be fitted quantitatively, but the slow build-up implies that the interaction between silicon and boron is weak or that only few silicon sites are in the close proximity of boron. (b) The spectra based on which REDOR fractions at a recoupling time of 7.68 ms were calculated. The REDOR fraction is calculated as $(S0 - S) \div S0$.

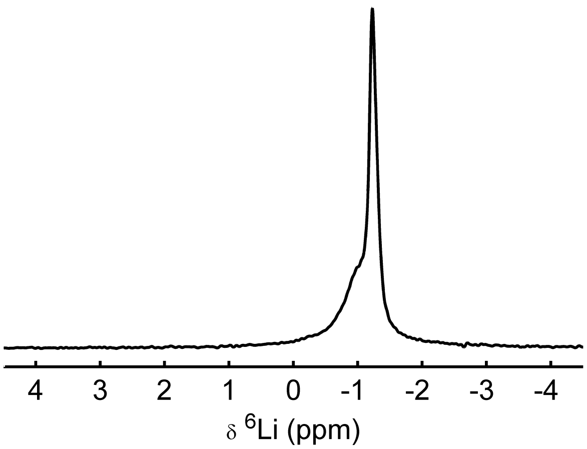

Figure S7: ^{6}Li NMR spectrum of the nanocomposite BM(Ar)-AS, measured at 22.3 T under 18 kHz MAS using a fixed recycle delay of 200 s. The spectrum consists of a broader peak around −1.0 ppm and a narrow peak around −1.2 ppm. This is consistent with the spectrum of melt-infiltrated nanocomposites below the structural phase transition.[S3]

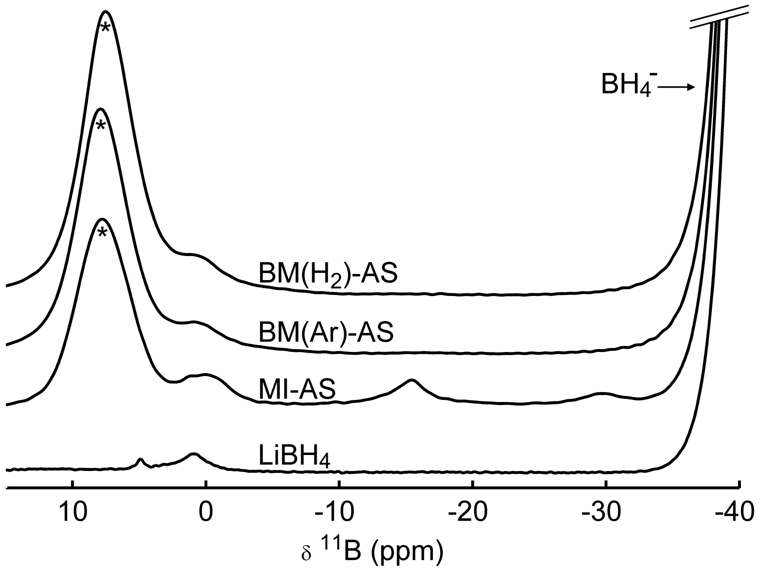

Figure S8: Magnified ^{11}B MAS NMR spectra of bulk $LiBH_4$ and nanocomposites with Aerosil, as shown in Figure 6a, revealing minor peaks for the melt infiltrated nanocomposite and peaks between 0 and 10 ppm present in all spectra including bulk $LiBH_4$. Spinning sidebands are indicated by asterisks; the spectrum of bulk $LiBH_4$ was spun faster and thus does not have spinning sidebands in this spectral region. The large, truncated peak on the right corresponds to the BH_4^- resonance of $LiBH_4$.

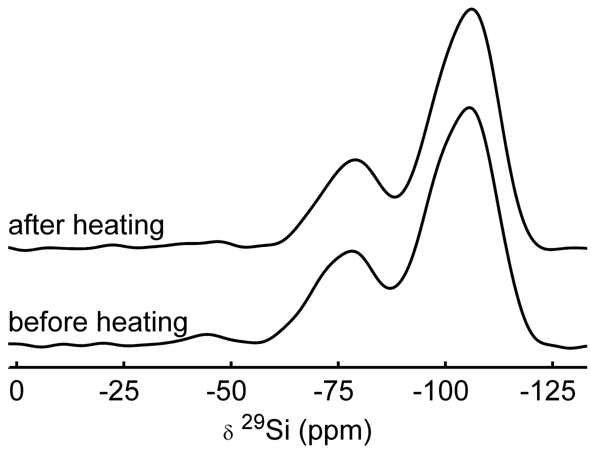

Figure S9: ^{29}Si CP NMR spectra of the nanocomposite BM(Ar)-SBA before and after being heated to 116 °C in N_2 for 80 min in steps of approximately 12 °C/h. The spectra were acquired at 7.05 T under 6.25 kHz MAS using CPMG detection.

References

(S1) Lambregts, S. F. H.; van Eck, E. R. H.; Ngene, P.; Kentgens, A. P. M. The Nature of Interface Interactions Leading to High Ionic Conductivity in $LiBH_4/SiO_2$ Nanocomposites. *ACS Appl. Energy Mater.* **2022**, *5*, 8057–8066.

(S2) Hirschinger, J. Analytical Solutions to Several Magic-Angle Spinning NMR Experiments. *Solid State Nucl. Magn. Reson.* **2008**, *34*, 210–223.

(S3) Lambregts, S. F. H.; van Eck, E. R. H.; Suwarno; Ngene, P.; de Jongh, P. E.; Kentgens, A. P. M. Phase Behavior and Ion Dynamics of Nanoconfined $LiBH_4$ in Silica. *J. Phys. Chem. C* **2019**, *123*, 25559–25569.
